# Increasing numbers of acute hepatitis C infections in HIV-infected MSM and high reinfection rates following SVR

**DOI:** 10.1186/1758-2652-13-S4-P200

**Published:** 2010-11-08

**Authors:** HJ Stellbrink, CK Schewe, M Vogel, C Noah

**Affiliations:** 1 ICH Study Center, Hamburg, Germany; 2 University Hospital, Medical Department, Bonn, Germany; 3 IPM Biotech, Hamburg, Germany

## Objective

To analyze the characteristics and outcome of acute Hepatitis C and the risk of reinfection following SVR in HIV+ MSM.

## Methods

Database analysis of the ICH cohort (n=4851), analysis restricted to MSM seen between 1/2002 and 6/2010. Acute HCV infection was defined as a sudden rise in liver enzymes with a positive HCV PCR and/or a newly positive HCV serology (if negative before). The probability of reinfection was analyzed from primary diagnosis or end of HCV treatment until reinfection or last f/u (last blood sample).

## Results

99 episodes of acute Hepatitis C (aHCV) were identified in 88 MSM since 2002. Case numbers increased since 2006, with no association with ART treatment status, HIV viral load or CD4 count. 42% had GT1a/b, 1% GT 2, 17% GT3, and 26% GT4. SVR was achieved in 45 (74%) of those 61 patients observed for more than 6 months after diagnosis or the end of HCV therapy either spontaneously (n=13) or after treatment with pegIFN/RBV (n=32). Reinfections occurred in 10 of these cases (11%), 3x with the same GT, 7x with a different GT (1x 1->3, 4x 3->1, 1x 4->1, 1x 1->4); reinfection was observed after spontaneous clearance in 4 and after treatment-induced clearance in 6 cases. 1 patient exhibited spontaneous clearance of two GT1a reinfections and an initial GT3 infection. In those achieving SVR, the cumulative probability of becoming reinfected after primary diagnosis or the end of HCV therapy, respectively, was 45% within six years (Kaplan-Meier). Within the time-frame of the analysis, only three cases of aHCV were observed in our STD clinic population in HIV-negative MSM (all GT4).

## Conclusions

Acute hepatitis C is an increasing problem in HIV+ MSM. The distribution of genotypes indicates an epidemic separate from the general population. Despite a high overall rate of spontaneous or treatment-induced SVR, patients are at high risk of reinfection with the same GT or others. These observations strongly support the EACS guidelines with regard to routine HCV testing and evaluation of LFT abnormalities. All HIV+ MSM, but especially subjects with previous aHCV should receive intensive and repeated counseling in order to reduce transmission risks.

